# Supplementary material for: Splice-Junction-Based Mapping of Alternative Isoforms in the Human Proteome
Source: Cell Rep. Author manuscript; Available in PMC 2020 Jan 15. (PMC6961840; doi:10.1016/j.celrep.2019.11.026)
Supplement: 2 [file NIHMS1546469-supplement-2.zip › DF1/PXD006675/VentricularSeptum/VentricularSeptum_35_PRPF40B_TLLQQLDDHGPMEPGIPPPFPPMGLPPMSQRP.pdf]

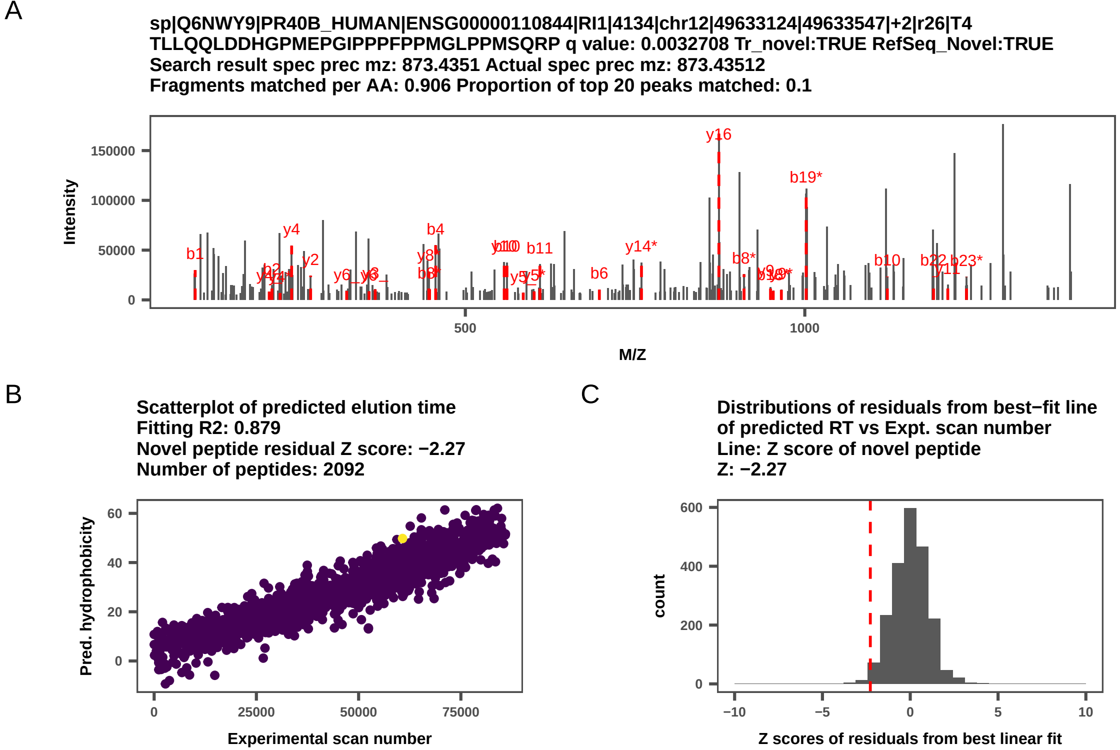
A
sp|Q6NWY9|PR40B_HUMAN|ENSG00000110844|RI1|4134|chr12|49633124|49633547|+2|r26|T4
TLLQQLDDHGPMEPGIPPPFPPMGLPPMSQRP q value: 0.0032708 Tr_novel:TRUE RefSeq_Novel:TRUE
Search result spec prec mz: 873.4351 Actual spec prec mz: 873.43512
Fragments matched per AA: 0.906 Proportion of top 20 peaks matched: 0.1
Intensity
150000
100000
50000
0
500
1000
M/Z
y16
b19*
y4
b4
b1
b11
y14*
y2
b6
b8*
b10
b22
b23*
y11
y6
B
Scatterplot of predicted elution time
Fitting R2: 0.879
Novel peptide residual Z score: −2.27
Number of peptides: 2092
Pred. hydrophobicity
60
40
20
0
0
25000
50000
75000
Experimental scan number
C
Distributions of residuals from best−fit line
of predicted RT vs Expt. scan number
Line: Z score of novel peptide
Z: −2.27
count
600
400
200
0
−10
−5
0
5
10
Z scores of residuals from best linear fit
